# Human–AI co-creation in generative AI video: content characteristics, flow experience, and continued creation intention—a hybrid SEM–ANN study

**DOI:** 10.3389/fpsyg.2026.1897624

**Published:** 2026-09-09

**Authors:** Min Hou, Jiayi Zhang, Mengyi Liu, Jing Pu

**Affiliations:** School of Arts and Media, Sichuan Agricultural University, Ya’an, China

**Keywords:** AI video creation willingness, content characteristics, flow experience, flow theory, generative AI video, SEM–ANN hybrid analysis, S-O-R framework

## Abstract

**Introduction:**

Grounded in the stimulus–organism–response (S-O-R) theory and flow theory, this study investigates the pathways through which five dimensions of artificial intelligence (AI) video content characteristics—generation quality, emotional resonance, visual novelty, interaction, and narrative coherence—influence university students’ AI video creation willingness (AW), with a particular focus on the mediating role of flow experience.

**Methods:**

A hybrid approach combining structural equation modeling (SEM) and artificial neural network (ANN) analysis was adopted. Using the Jimeng (Dreamina) AI video generation platform as the research context, questionnaire data were collected from 512 Chinese university students with more than 1 month of usage experience.

**Results:**

All five AI video content characteristics significantly and positively influenced flow experience, which in turn significantly affected AW. Among these characteristics, visual novelty and narrative coherence exerted the most prominent effects on AW. Bootstrap mediation tests confirmed that flow experience significantly mediated all examined pathways. ANN sensitivity analysis further verified that visual novelty was the primary predictor of flow experience, while flow experience was the core predictor of creation willingness.

**Discussion:**

This study extends the application boundary of S-O-R theory to generative AI creation contexts and elucidates the psychological mechanism through which external stimuli are transformed into behavioral responses. The findings also provide theoretical grounding and practical guidance for AI video platforms to optimize user experience and enhance users’ willingness to adopt AI video creation.

## 1 Introduction

Generative artificial intelligence (AI) has fundamentally reshaped digital media production, shifting creative practice from single-technology operation toward human–AI collaboration. In AI video generation, users are no longer passive viewers of pre-made content; they participate in creation through prompt input and content regeneration. AI video generation, therefore, should be understood as a process of human–AI co-creation. AI video content characteristics refer to generation quality, emotional resonance, visual novelty, interaction, and narrative coherence (Yoo et al., 2012). Currently, AI video generation is primarily realized through website platforms, but its content characteristics differ fundamentally from those of traditional media. Traditional media content is pre-produced and passively consumed, whereas AI video content draws users deeply into the creative process through prompt input and iterative refinement. This difference calls for a contextualized recalibration when moving the stimulus–organism–response (S-O-R) theory from static consumption settings to dynamic creation settings (Jacoby, 2002). Compared with traditional website platforms that mainly focus on information display and transactional functions, AI video platforms place greater emphasis on users’ creative expression and immersive experiences, exhibiting pronounced visual novelty and high interaction (Cao et al., 2023). The university student population demonstrates high technological sensitivity and strong creative demand. Studies indicate that approximately 62% of university student users regard AI video output content as an important tool for self-expression and leisure entertainment (Yu et al., 2024). However, related research also points out that the activity decline rate among university student users reaches 47% after 3 months of use (Rezwana and Maher, 2023), highlighting the prominent issue of their low sustained creation willingness. Therefore, this study adopts the human–AI collaboration perspective as an overarching theoretical lens (Rezwana and Maher, 2023), extends the traditional S-O-R framework, and explains how AI video content characteristics shape university students’ immersive experience and sustained creation willingness.

Existing research has predominantly examined the mechanisms influencing sustained creation willingness from the perspectives of content perceived quality and interaction participation characteristics (Bellary et al., 2024; Lee and Kim, 2017). For instance, studies have explored the effects of generation quality, emotional resonance, and visual novelty on continuance adoption (Tarafdar and Zhang, 2005); as well as the effects of interaction, emotional resonance, and generation quality on self-immersion (Jin et al., 2023). However, these studies exhibit three limitations: First, existing research largely treats AI video platforms as technical tools or content platforms, without fully explaining the psychological mechanisms underlying users’ engagement with AI-generated video content. Second, prior S-O-R studies mostly stem from static media consumption or website usage, without adequately addressing how the meaning of the S-O-R theory shifts in the context of AI video generation. Third, existing research predominantly employs linear models, making it difficult to capture the complex predictive relationships inherent in AI video generation. Addressing these research gaps, this study proposes the following innovations in the context of AI video creation: First, introducing flow theory into the study of AI video content characteristics to explore university students’ sustained creation willingness under flow states. Second, adopting a hybrid approach combining structural equation modeling (SEM) and artificial neural network (ANN) to both validate linear relationships among variables and capture potential nonlinear effects, thereby enhancing the robustness of research findings. Third, constructing a five-dimensional analytical framework of AI video content characteristics to systematically elucidate their influence mechanisms on flow experience and sustained creation willingness.

Building on this, this study takes university students as the research subjects and investigates the structural relationships among the five dimensions of AI video content characteristics, flow experience, and creation willingness, with a particular focus on revealing the mediating role of flow experience and the mechanisms through which each variable exerts influence. The specific research questions are as follows:

How do the five content characteristics of AI video platforms influence university students’ AI video creation willingness (AW) through the psychological mechanism of flow experience?

In the context of AI video generation, do visual novelty and narrative coherence exhibit higher relative importance in predicting flow experience and AW?

Are the SEM path coefficients and ANN sensitivity indices highly consistent in ranking the importance of AI video content characteristics?

## 2 Theoretical foundations and research hypotheses

In this section, a new conceptual model that explores the influence of AI video content characteristics on university students’ AW is introduced, following a discussion of the relationships among the relevant variables.

### 2.1 Flow experience and S-O-R theory

Flow experience, also known as flow theory, was first proposed by American psychologist Csikszentmihalyi M. He discovered that different interviewees experienced a remarkably similar “optimal experience” (Csikszentmihalyi, 2000) when engaging in corresponding activities. In the field of AI video content characteristics, flow experience refers to the state in which university students, through interface interaction and real-time feedback from AI-generated video content, gradually enter a highly focused and creativity-arousing immersive state, thereby obtaining physical and mental pleasure (Novak et al., 2003). Meanwhile, flow experience can reflect university students’ cognitive and emotional states, potentially influencing their behavioral responses and even correlating with their usage behavior process (Dwivedi et al., 2023). The more prominent the visual novelty design of AI video content characteristics, the stronger the university students’ flow experience (Cuny, 2015), thereby enhancing their creation willingness. Many scholars have explored the significant influence of flow experience on university students’ AW (Pelet et al., 2017). For example, Gu found that students’ flow experience influences their continuance learning intention in the study of digital painting (Gu et al., 2022). Some scholars have shown that flow theory is positively correlated with AW in VR research (Hassan et al., 2020). In summary, the impact of flow experience on AW has been validated in digital art platforms. As AI video content characteristics belong to a type of digital art platform, in the usage context of AI video content characteristics, flow experience will still influence university students’ AW.

Mehrabian and Russell proposed the “stimulus-organism-response” (S-O-R) theory (Vieira, 2013). In this study, AI video content characteristics are regarded as stimulus factors, acting on university student users through dimensions such as visual presentation, interaction, and emotional resonance. Flow experience serves as the core organism factor, reflecting the highly focused and pleasurable psychological state that university students achieve during the AI video creation process; AW constitutes the individual response factor, embodying university students’ behavioral intention driven by specific stimuli and psychological experiences (Bai et al., 2024). This theory has good theoretical adaptability in explaining the influence of interaction and emotional resonance on AW (Kumar et al., 2021). Previous studies have verified the effectiveness of the S-O-R theory in research on continuance intention to use social platforms (Feng, 2024), laying an empirical foundation for the hypotheses proposed in this study.

From the human–AI collaboration perspective, whether S-O-R and flow theories can effectively explain AI video content characteristics depends on how well the core assumptions of these two frameworks match the contextual features of this setting (Bigne et al., 2020). This study contends that both theories hold explanatory potential but require contextual adaptation to address the specificities of AI video generation. Specifically, in traditional S-O-R research, external stimuli mostly take the form of static environmental cues, whereas in AI video generation, content quality continuously evolves through real-time user–platform interaction, and users’ prompt inputs themselves constitute new sources of stimulation (Cao et al., 2023). Traditional research on organism states has largely focused on singular affective responses, yet AI video creation demands that flow experience be defined as a composite state in which users simultaneously experience heightened concentration, temporal distortion, creative enjoyment, and enhanced self-efficacy during the creative process (McGuire et al., 2024)–a complex psychological state that cannot be captured by conventional unidimensional organism variables. Behavioral responses in traditional studies are often one-time decisions, whereas AW is cumulative and iterative: the positive experiences users accumulate during use convert into sustained creation willingness, and the act of creation itself, in turn, reshapes users’ perceptions of the platform’s content characteristics. Moreover, while existing S-O-R empirical studies commonly employ SEM to test linear paths, scholars who have revised the S-O-R framework have pointed out that simple linear sequences may obscure dynamic relationships. AI video output is also subject to prompts, random seeds, and parameter settings, introducing stochasticity and nonlinearity; SEM-ANN can simultaneously handle theoretical path testing and nonlinear relationships (Pholsook et al., 2023). From this perspective, S-O-R theory and flow theory provide a suitable mechanism for explaining how AI-generated video content elicits users’ psychological responses and behavioral intentions.

### 2.2 AI video content characteristics

AI video content characteristics differ fundamentally from those of traditional media, and this difference constitutes the core basis for theoretical transfer. Traditional media content is pre-produced and passively consumed; user–content interaction is limited to playback control, and content quality is fixed before consumption. In contrast, AI video content is generated in real time and co-created actively; users participate deeply in the content production process through prompt input and other means, and content quality emerges dynamically through interaction. This difference demands that the measurement of AI video content characteristics must encompass AI-specific dimensions such as generation quality, interaction, and visual novelty. In analyzing designers’ AIGC usage behavior, Wang and Chen pointed out that the stimulus dimensions of AIGC tools should cover multiple facets including system quality, information quality, service quality, and hedonic value (Wang and Chen, 2024); the five-dimensional framework of this study represents a concretization and contextualization of this approach.

AI video content characteristics possess the dual attributes of creation tools and leisure entertainment, necessitating a multidimensional framework for interpretation. Emotional resonance refers to the degree of fit between AI-generated content and users’ emotional needs, that is, whether AI-generated video content can trigger users’ emotional responses, value identification, and psychological connection. Visual novelty can strengthen sensory immersion, enabling aesthetic stimuli to enhance university students’ emotional responses (Coyle and Thorson, 2001); interaction supports real-time human–computer dialogue, facilitating bidirectional interaction between AI video content characteristics and university students, thereby enhancing their flow experience (Novak et al., 2000); narrative coherence, by constructing logically complete and plot-coherent story structures, reduces users’ cognitive load and strengthens their sense of creative control, promoting deep immersion (Zhou and Qu, 2026).

### 2.3 Research hypotheses

#### 2.3.1 Emotional resonance, interaction, generation quality, visual novelty, narrative coherence, and flow experience

From the human–AI collaboration perspective, flow experience does not arise from merely viewing content; rather, it emerges from a process in which the user’s intentions are understood, translated, fed back, and readjusted by the system. Content characteristics that enhance emotional alignment, interactive control, quality realization, visual surprise, and narrative continuity will therefore strengthen users’ sense of focus, control, and enjoyment during the creative process.

Emotional resonance facilitates the formation of flow experience through emotional engagement. From the human–AI collaboration perspective, emotional resonance originates from the emotional expression and aesthetic experience carried by AI-generated video content itself (Niu et al., 2025). When AI-generated video content accurately captures and amplifies users’ emotional intentions, users experience emotional resonance, a state characterized by highly focused attention and the temporary weakening of self-awareness (Goldenberg and Gross, 2020), thereby becoming a fundamental driver of flow experience.

Interaction fosters university students’ flow experience through real-time feedback. On traditional platforms, interaction largely involves information retrieval or social exchange, whereas in AI video generation contexts, interaction is iterative: users input prompts, the system generates videos, users evaluate the output and adjust prompts, and the system regenerates. This loop aligns users’ actions with system responses toward creative goals, thereby inducing flow experience. Hoffman and Novak pointed out that highly interactive media environments induce flow experience by aligning users’ actions with immediate system responses (Hoffman and Novak, 1996). Therefore, when the interface of AI video content characteristics operates smoothly, can quickly respond to university students’ intentions, and provides timely feedback on the results they need, university students are able to obtain flow experience through continuous interaction.

Generation quality, as the embodiment of functional value, directly correlates with users’ actual need fulfillment and serves as the foundational guarantee for sustained creation willingness. In traditional video production, quality improvement depends on professional equipment and accumulated skills, whereas AI video platforms enable users to obtain high-quality output at low cost through algorithmic iteration. Kulichyova et al. found that generative AI acts as a cognitive and visual scaffold, accelerating users’ creative ideation process; the capability leap that university students experience through AI assistance directly translates into creative confidence and sustained engagement (Gu, 2026). Therefore, when the videos generated by the AI video platform meet or exceed user expectations in terms of resolution, motion coherence, and semantic consistency, users not only satisfy their immediate creative needs but also strengthen their long-term interest through the sense of achievement brought by technological empowerment.

Visual novelty promotes flow experience through multisensory stimulation and aesthetic appeal. From the human–AI collaboration perspective, visual novelty, through algorithmically generated unpredictable elements such as style transfer and surreal scenes, can stimulate university students’ aesthetic pleasure and sustained creation willingness. Coyle and Thorson demonstrated that visually rich interfaces and dynamic media elements can capture attention and elicit emotional responses (Coyle and Thorson, 2001). Neuroscientific research further indicates that vivid sensory input activates brain regions associated with pleasure and concentration, thereby enhancing university students’ engagement with AI video content characteristics (Brasel and Gips, 2014). Therefore, when the interface of AI video content characteristics is exquisitely designed and the visuals are vivid, university students are able to obtain flow experience.

Narrative coherence drives flow experience through storytelling. The narrative logic of AI video generation differs from that of traditional video production. Narrative coherence in AI video generation is inherently constrained by the algorithm’s comprehension capacity; the ambiguity of prompts and the randomness in the generation process can both lead to narrative discontinuity. Yet it is precisely this limitation of algorithmic comprehension that opens up participatory space for university students to complete the narrative. When perceiving minor narrative incoherence, university students actively restore narrative consistency by adjusting prompts, regenerating content, or imposing new interpretive frameworks on the output—and this completion process itself becomes a source of deep flow experience. In digital art contexts, interactive narratives enhance university students’ sustained participation by triggering dopamine release (Hamari et al., 2016). Thus, a coherent narrative structure can reduce cognitive load, enabling university students to enter a deep flow state of “losing track of time”.

Based on the above analysis, the following hypotheses are proposed:

*H1*: Emotional resonance has a significant positive impact on flow experience.

*H2*: Interaction has a significant positive impact on flow experience.

*H3*: Generation quality has a significant positive impact on flow experience.

*H4*: Visual novelty has a significant positive impact on flow experience.

*H5*: Narrative coherence has a significant positive impact on flow experience.

#### 2.3.2 Emotional resonance, interaction, generation quality, visual novelty, narrative coherence, and AI video creation willingness

In AI video generation platforms, sustained creation willingness is not simply the continued use of a technology; rather, it is the user’s willingness to re-engage in the cyclical process of prompt design, result selection, content revision, and work publication. When users perceive that AI can effectively support their expression, control, aesthetic exploration, and narrative construction, they are more likely to develop sustained creation willingness.

Emotional resonance directly drives sustained creation willingness by constructing emotional bonds between users and the platform. In traditional media, emotional connection stems from users’ passive identification with content, whereas in AI video generation, emotional resonance arises from the process in which users’ actively input emotional intentions are accurately restored and amplified by AI (Wang and Yang, 2024). When users express emotional needs through prompts, the AI video platform translates them into corresponding visual narratives; in the experience of seeing their intentions transformed into reality, users establish a deep emotional connection with the AI video platform. This connection not only enhances immediate creation satisfaction but, through the accumulation of emotional memory, converts into sustained creation willingness. Specifically, emotional resonance directly drives sustained creation willingness by constructing emotional bonds between users and the platform (Zhang et al., 2025).

Interaction reinforces and consolidates sustained creation willingness through dynamic feedback mechanisms that deepen engagement and belonging. Interaction on traditional platforms largely involves information retrieval or social exchange, whereas interaction on AI video platforms is an iterative dialogue centered on creative goals. This loop allows users to progressively approach their ideal output through repeated adjustments, with each iteration strengthening users’ dependence on the platform. The “short video” section in AI video content allows users to draw inspiration from each other, forming a benign competitive atmosphere. This social motivation enables university students to experience collective recognition during creation, thereby becoming more inclined to continue creating (Coyle and Thorson, 2001).

Generation quality, as the embodiment of functional value, directly correlates with the degree of users’ actual need fulfillment and serves as the foundational guarantee for sustained creation willingness. In traditional video production, quality improvement depends on professional equipment and accumulated skills, whereas AI video platforms enable users to obtain high-quality output at low cost through algorithmic iteration. When the videos generated by the platform meet or exceed user expectations in terms of resolution, motion coherence, and semantic consistency, users not only satisfy their immediate creative needs but also strengthen their long-term interest through the sense of achievement brought by technological empowerment (Liu et al., 2024).

Visual novelty enhances sustained creation willingness by stimulating emotional resonance through sensory stimulation and aesthetic design. In the context of AI video generation, visual novelty exhibits the dual characteristics of technological democratization and social display. On the one hand, AI algorithms empower ordinary users to achieve advanced visual expression without professional training through techniques such as style transfer and surreal scene generation (Coyle and Thorson, 2001); this leap in creative capability directly translates into a sense of achievement and sustained creative motivation. On the other, when users share surreal videos on social platforms, the irreproducible nature of such content garners higher attention and recognition, further consolidating sustained creation willingness.

Narrative coherence drives sustained creation willingness through storytelling. Continued use of traditional video platforms often relies on external rewards, whereas sustained creation willingness on AI video platforms stems from users’ intrinsic motivation to actively construct a personal narrative system. When users find that the platform can stably generate logically complete and plot-coherent stories, they tend to continue creating around specific themes or characters, transforming from one-time creators into long-term narrative builders. Moreover, the algorithmic variation in character behavior within AI-generated narratives provides unexpected surprises for story development; this controlled unpredictability maintains creative freshness while stimulating the desire to explore further narrative possibilities.

In summary, AI video content characteristics influence university students’ AW to varying degrees. Whether university students can continue using AI video generation platforms mainly depends on whether the AI video content characteristics can meet their expectations and achieve their satisfaction, thereby enhancing AW.

Based on the above analysis, the following hypotheses are proposed:

*H6*: Emotional resonance has a significant positive impact on AW.

*H7*: Interaction has a significant positive impact on AW.

*H8*: Generation quality has a significant positive impact on AW.

*H9*: Visual novelty has a significant positive impact on AW.

*H10*: Narrative coherence has a significant positive impact on AW.

#### 2.3.3 Flow theory and AI video creation willingness

Flow experience refers to a subjective feeling of “complete engagement” and “selfless obsession” that individuals experience when performing certain activities. Characterized by deep enjoyment, it is influenced by personal, environmental, and situational factors (Teng, 2011). Personal traits such as clear goals, skills, confidence, and intrinsic motivation facilitate university students’ entry into a flow state. Interaction also promotes users’ flow experience (Novak et al., 2003). When university students are engaged in such “immersive” experiences, they are able to feel a sense of pleasure and satisfaction beyond what they ordinarily experience (Csikszentmihalyi, 2000).

From the above, it can be seen that the strong interaction and generation quality of AI video content characteristics enable university students to become fully immersed, thereby influencing their AW (Vittersø et al., 2001; Hsu et al., 2012). For instance, Yoon and Son (2021) evaluated the effects of immersion experience on museum visitors’ continuance intention to visit; some scholars have demonstrated that flow experience positively influences university students’ AW (Feng et al., 2019). Therefore, when university students deeply engage with AI video content characteristics, they experience psychological and physical pleasure as well as a sense of achievement, which can better stimulate their motivation to continue using AI video. Flow experience is critical in determining university students’ satisfaction. A favorable flow experience is more effective in inducing university students’ motivation and capability, thereby triggering their AW (Inal, 2023; Inal, 2023).

Based on the above analysis, the following hypothesis is proposed:

*H11*: Flow experience has a significant positive impact on AW.

#### 2.3.4 Mediating role of flow experience

Flow experience is often regarded as a key mediating variable connecting university students’ perceived characteristics with their behavioral decisions. Guo and Barnes (2007) virtual reality research demonstrated that flow experience indirectly enhances A by strengthening users’ perceptions of content emotional resonance and generation quality. A recent study examined the mediating role of flow experience in live-streaming commerce (Joo and Yang, 2023), while Hewei, T. et al. confirmed its mediating effect in the relationship between social media interaction and AW (Hewei and Youngsook, 2022). These findings not only reveal the direct impact of flow experience on AW but also highlight its mediating mechanism, providing theoretical support for university students’ sustained use of AI video content characteristics. Thus, we can reasonably propose the following hypothesis:

*H12*: Flow experience mediates the effect of AI video content characteristics on university students’ AW.

**Figure 1:**
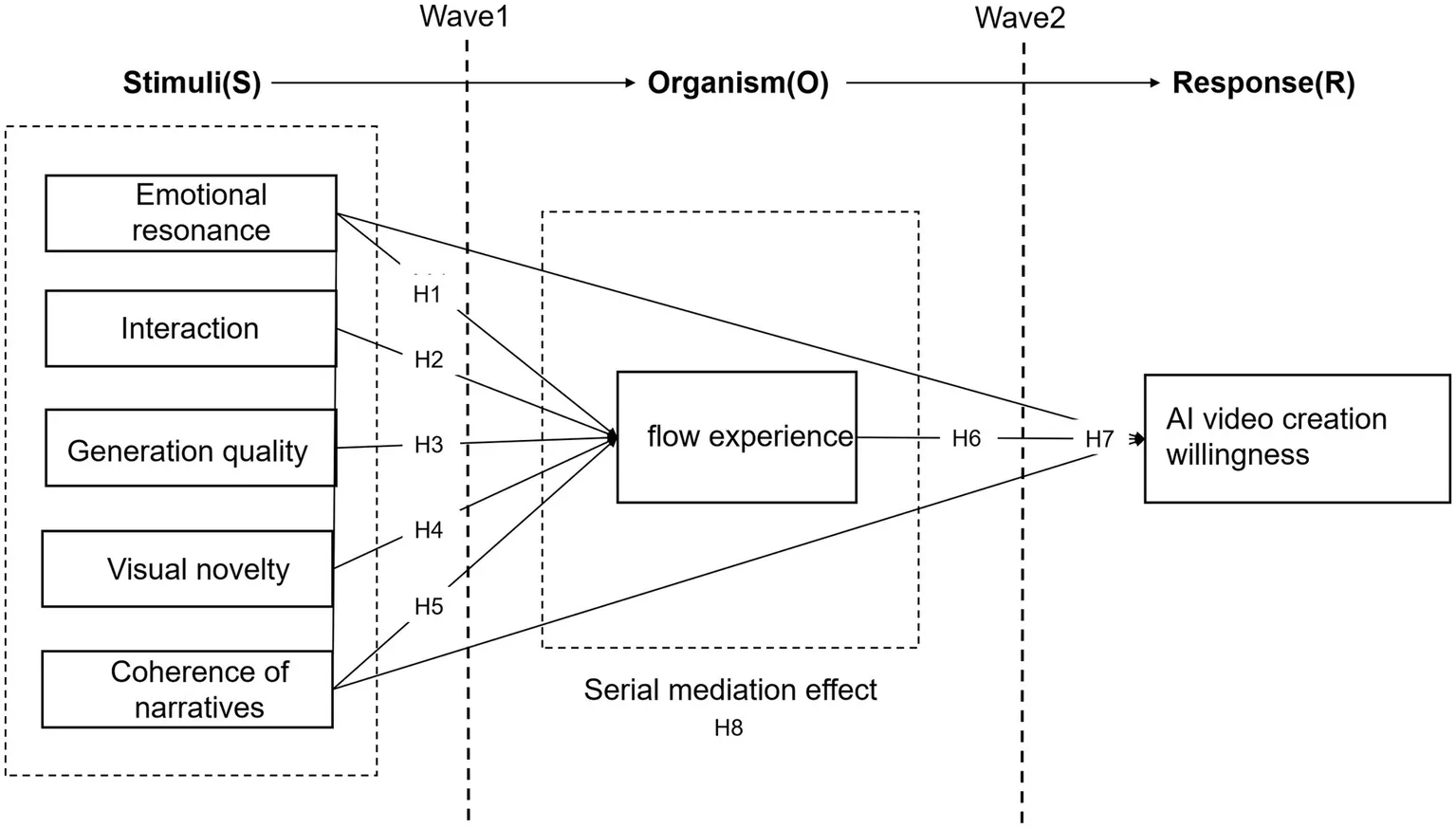
Conceptual model.

Finally, this study establishes a theoretical model (Figure 1) that examines the influence of AI video content characteristics on university students’ AW, with flow theory as the mediating framework.

## 3 Research methodology and experimental design

### 3.1 Experimental preparation

A survey-based design was adopted for this study. All procedures were conducted in accordance with the Declaration of Helsinki and relevant institutional guidelines and regulations. The study protocol was approved by the Ethics Committee of the School of Arts and Media, Sichuan Agricultural University (approval number: IRB-2024-06-17). Informed consent was obtained from all participants prior to the survey. Participation was entirely voluntary, anonymity was ensured, and participants retained the right to withdraw at any time without penalty. After reviewing these terms, each participant was asked to actively select “I agree to participate.” This process guaranteed voluntary, informed participation and an explicit right to withdraw at any point.

To control for differences in technological proficiency, all participants received standardized operational training on the AI video generation platform Jimeng (Dreamina). The training covered platform interface navigation, an introduction to basic text-to-video and image-to-video functions, fundamental prompt-writing skills, video generation parameter settings, and a hands-on practice task in which participants generated a 15-s short video. Researchers provided on-site technical support and answered operational questions but refrained from evaluating the creative content, so as not to exert psychological pressure on participants. The practice task was limited to 10 min; participants who did not complete it within that time still proceeded to the subsequent formal experiment.

### 3.2 Participants and experimental procedure

**Figure 2:**
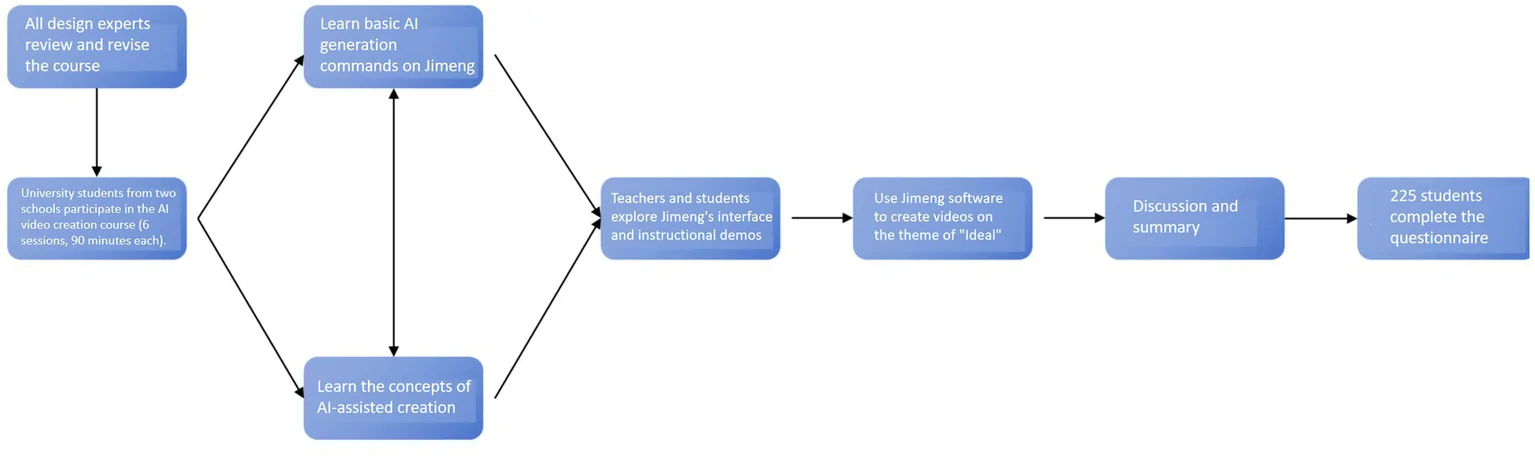
The experimental process.

The data for this study were drawn from both offline experimental samples and online supplementary samples. For the offline experiment, a convenience sampling method was employed to recruit 368 Chinese students from various academic years and majors across two universities in southwest China. Among them, 200 were female (54.3%) and 168 were male students (45.7%). This sampling method was chosen for its practicality and accessibility, facilitating the efficient recruitment of participants from the target population. Demographic details including gender and income were collected to provide insights into the sample profile, allowing for a more nuanced analysis of the findings. The students participated voluntarily in the research experiment and were fully informed about the procedures. They enrolled in a one-month course on AI video creation, primarily focused on learning about AI video generation using the Jimeng (Dreamina) tool. The core content of this course was AI video generation creation. All students who attended the course offline were those with no prior experience in AI video generation. The experimental process is illustrated in Figure 2.

The course was implemented over 1 month, with one session per week for 4 weeks, dedicated to creating AI video generation works themed “Ideal.” The course design is detailed in Table 1. From Session 2 to Session 3, students delved into using Jimeng (Dreamina) to produce their AI videos.

**Table 1:** Course design.

Session	Activities
1	Understanding and learning the concepts of AI video creation, gaining a concrete understanding of AI video production, and setting the theme of this activity as “Ideal.”
	What is AI video creation?
	The context of AI video creation.
	Learning the AI video creation course and basic commands through the AI video generation website Jimeng (Dreamina).
2–3	The instructor explains to the students how to write a script and the fundamentals of camera work.
	The instructor demonstrated how to use Jimeng (Dreamina) to create videos with a theme.
	Students discussed to determine their video topics and then used Jimeng (Dreamina) to create their videos. Students and the instructor shared and communicated to solve problems encountered during creation.
4	Upon completion of the assignment, the instructor and students communicated to discuss gains and issues identified during the course.
	Finally, course feedback was completed, with students filling out the AI video generation questionnaire.

### 3.3 Questionnaire sample and data collection

Prior to the formal survey, we conducted a small-scale pilot test with 100 participants. Both the offline and online questionnaires underwent the same pilot procedure as the formal survey, and 90 valid questionnaires were ultimately recovered. The collected data were subsequently analyzed, with the following results: the Cronbach’s alpha coefficient was 0.836, the Kaiser–Meyer–Olkin (KMO) value was 0.724, the approximate chi-square value of the Bartlett’s test of sphericity was 2671.579, and the significance level was 0.000. These values met the established criteria, confirming that the questionnaire successfully passed both reliability and validity assessments.

After the course, we conducted the formal survey, with students voluntarily completing an anonymous questionnaire. The online survey was administered through the Chinese online platform “Question Star.” The online recruitment targeted university students with no prior experience using AI video generation websites, who completed a one-month online learning program by watching recorded lectures. After completing their learning, the online questionnaire was distributed via popular online forums and social networking sites, and the link to the online questionnaire was sent to respondents in early January 2026. By the end of February 2026, a total of 546 questionnaires were collected from both offline and online sources. Following a screening process, responses from participants who had no experience using AI video generation websites, those who completed the survey in less than 1 minute, and those who selected identical options throughout the questionnaire were excluded. As a result, 512 valid questionnaires were retained, yielding an effective response rate of approximately 95.8%. Among the valid responses, analysis by gender revealed that female participants slightly outnumbered male participants, accounting for 55.66 and 44.34%, respectively. Regarding income status, the “unemployed” group constituted the highest proportion. Detailed information is provided in Table 2.

**Table 2:** Frequency analysis results.

Name	Option	Frequency	Percentage (%)
Gender	Male	227	44.34
	Female	285	55.66
Grade	Freshman	39	7.62
	Sophomore	49	9.57
	Junior	67	13.09
	Senior	73	14.26
	Master	80	15.63
	PhD	87	16.99
	Employed	117	22.85
Income	Unemployed	395	77.15
	2,000–4,000	29	5.66
	4,000–8,000	51	9.96
	Above 10,000	37	7.23

### 3.4 Questionnaire design and measurement

All constructs in this study were measured using validated multi-item scales adapted from prior literature in media psychology and human–computer interaction. All items were rated on a five-point Likert scale (1 = strongly disagree, 5 = strongly agree). The five content characteristic dimensions served as stimulus variables, comprising interaction, emotional resonance, visual novelty, generation quality, and narrative coherence. These constructs were adapted from traditional media content evaluation literature but were redefined to account for the probabilistic and generative nature of AI video content.

Interaction quality refers to users’ perceived responsiveness and controllability during the human–AI co-creation process in AI video generation.

Narrative coherence refers to the perceived temporal, semantic, and causal consistency of multi-shot sequences dynamically generated by stochastic models under user control. Unlike traditional scripted media, coherence in AI video generation is not determined by predefined storylines but arises from the iterative alignment between user prompts and model outputs.

Visual novelty refers to the unexpected yet meaningful visual variations produced by AI systems through the probabilistic recombination of learned visual patterns. In the context of AI video generation, visual novelty is defined not merely by originality but by the degree to which outputs deviate from users’ prior expectations while maintaining semantic interpretability.

Emotional resonance reflects the extent to which AI-generated video content evokes users’ emotional responses and affective engagement. This construct remains conceptually consistent with prior media psychology research but is shaped by the dynamic and personalized nature of machine-generated content (Çalli and Alma Çalli, 2025).

Generation quality refers to users’ evaluation of the technical and semantic quality of AI video output, encompassing visual fidelity, temporal consistency, and structural integrity. Unlike traditional media production quality, generation quality in AI systems is jointly determined by model capability, prompt specificity, and the stochastic variation inherent in output generation.

These adaptations ensure that the scale captures users’ perception of AI video-generated content as a co-created, dynamic, and stochastic process rather than a static media artifact. Thus, while the measurement model reflects the perceptual structures of traditional media, the existing scales have been contextually rewritten to tentatively capture user perceptions in AI video generation contexts; further development of dedicated scales remains warranted.

The specific scale items are presented below.

## 4 Data analysis

This study conducted a hybrid structural equation modeling—artificial neural network (SEM-ANN) analysis. This framework integrates the theoretical verification strengths of SEM with the pattern recognition capabilities of ANN, forming a complementary methodological design (Leong et al., 2020). Specifically, the SEM stage focuses on testing hypothesized linear relationships, validating the theoretical model through path analysis; the ANN stage breaks through the constraints of linear assumptions, utilizing a multilayer perceptron architecture to capture nonlinear interaction effects and complex decision-making patterns (Chong, 2013). This approach ensures the rigor of theory-driven inquiry while enhancing the model’s explanatory power regarding the complexity of real user behavior.

### 4.1 Common method Bias test

Given that all variables in this study were measured using self-report data collected through a single questionnaire at a single time point, common method bias (CMB) may be a concern. To mitigate this potential issue, multiple statistical procedures were employed. First, Harman’s single-factor test was conducted using exploratory factor analysis. The results showed that the first factor accounted for less than 40% of the variance, indicating no substantial common method bias. Second, a full collinearity assessment was performed based on the variance inflation factor (VIF). The VIF values for all latent variables were below 3, further suggesting that common method bias was not a serious concern in this study. Although a marker variable test was not adopted, prior research has demonstrated that combining Harman’s single-factor test with the full collinearity VIF approach can effectively detect potential CMB risks, thereby enhancing the robustness of the results (Kock, 2015). Taken together, these findings indicate that common method bias is unlikely to have materially affected the study’s conclusions.

### 4.2 Reliability and validity test

SPSS 26.0 was used to calculate the overall reliability of the questionnaire (see Table 3). The Cronbach’s α values for all seven variables exceeded 0.8, indicating high internal consistency. Additionally, the composite reliability for each variable was above 0.7, suggesting good overall reliability of the questionnaire. AMOS 24.0 was employed to fit the theoretical model, with confirmatory factor analysis used to assess convergent validity. The average variance extracted (AVE) and composite reliability (CR) values were evaluated to gauge convergent validity, while discriminant validity was assessed by comparing the square root of AVE with the correlation coefficients between variables. As shown in Table 4, the AVE values for all variables were above 0.5, and the CR values were above 0.8, indicating good convergent validity. Furthermore, as presented in Table 4, the square roots of the AVE values were greater than the absolute values of the inter-factor correlation coefficients, suggesting strong discriminant validity among the variables. In summary, both the reliability and validity of the questionnaire were confirmed, the measurement validity supports the theoretical transfer, and the subsequent hypothesis testing can proceed.

**Table 3:** Measurement scale.

Variable	Measurement item	Reference
Interaction (INT)	INT1: The AI video generation website responds quickly to my requests	Chiew and Salim (2003) Liu (2003) Alenezi et al. (2010)
	INT2: The AI video generation website provides useful and relevant information	
	INT3: I can share my views and exchange ideas with others on the AI video generation website	
	INT4: I can contact the website at any time to provide feedback or suggestions	
Emotional resonance (ER)	ER1: When using the AI video generation website, the generated video content can touch my emotions.	Lee et al. (2003) Yousafzai et al. (2007) Chiew and Salim (2003)
	ER2: The content of the AI video generation website makes me feel emotional resonance and connection.	
	ER3: The video content generated by the platform aligns with my emotional needs and mood state.	
	ER4: When using the AI video generation website, I can perceive the emotional value conveyed by the content.	
Visual novelty (VN)	VN1: I find the content on the AI video generation website lively and engaging	Zhang et al. (2021)
	VN2: The content on the AI video generation website is rich and varied	
	VN3: The background of the AI video generation website is visually appealing with vibrant colors	
Generation quality (GQ)	GQ1: The AI video generation website covers a wide range of topics that meet my needs	Lee et al. (2003) Venkatesh and Davis (2000) Chatterjee (2021)
	GQ2: The AI video generation website demonstrates a high level of professionalism	
	GQ3: The content and tools on the AI video generation website facilitate my work	
	GQ4: The AI video generation website is useful for my tasks	
Coherence of narratives (NC)	NC1: The AI video generation website shares detailed story content with you in an interesting way.	Taheri (2024)
	NC2: The AI video website can generate highly coherent storylines.	
	NC3: The content and plots provided by the AI video generation website are very vivid and engaging.	
Flow experience (FE)	FE1: The operations and responses while using the AI video generation website are smooth	Jennett et al. (2008)
	FE2: I know how to easily find the content I need on the AI video generation website	
	FE3: I enjoy using the AI video generation website so much that time passes quickly	
	FE4: I am fully immersed in my tasks on the AI video generation website	
AW	AW1: I am willing to adopt videos from the AI video generation website	Taheri (2024)
	AW2: I will pay attention to updates on the AI video generation website	
	AW3: I would recommend the AI video generation website to others	
	AW4: I would consider upgrading user level or purchasing a membership for a better experience	

**Table 4:** Reliability and validity analysis.

Variable	Item	CITC	Estimate	AVE	CR	α
AW	AW1	0.706	0.777	0.593	0.854	0.852
	AW2	0.671	0.743			
	AW3	0.707	0.789			
	AW4	0.691	0.771			
INT	INT1	0.649	0.732	0.564	0.838	0.837
	INT2	0.685	0.770			
	INT3	0.649	0.728			
	INT4	0.691	0.777			
ER	ER1	0.738	0.743	0.613	0.863	0.863
	ER2	0.665	0.789			
	ER3	0.709	0.771			
	ER4	0.729	0.732			
VN	VN1	0.650	0.770	0.586	0.809	0.809
	VN2	0.657	0.728			
	VN3	0.668	0.777			
GQ	GQ1	0.686	0.743	0.568	0.840	0.839
	GQ2	0.659	0.789			
	GQ3	0.633	0.771			
	GQ4	0.709	0.732			
NC	NC1	0.707	0.770	0.610	0.824	0.823
	NC2	0.671	0.728			
	NC3	0.660	0.777			
FE	FE1	0.708	0.743	0.631	0.872	0.872
	FE2	0.711	0.789			
	FE3	0.750	0.771			
	FE4	0.737	0.732			

### 4.3 Structural equation model test

To validate the 12 hypotheses, this study used AMOS 24.0 to test the structural model. The influence paths between variables were specified based on the theoretical model, and model fitting was conducted. As shown in Table 5, the CMIN/DF value was 1.281 (below the threshold of 3); the RMSEA was 0.023 (below the standard cutoff of 0.08), indicating a good fit; the GFI, IFI, and CFI values were 0.950, 0.988, and 0.988, respectively, all meeting the excellent fit criteria; and the PNFI value was 0.811 (above 0.5). All goodness-of-fit indices met the recommended thresholds, indicating that the model fits the data well. The results show that the proposed measurement model achieved an acceptable level of fit. The measurement model is thus valid in the context of AI-generated video content. The results of the hypothesis testing are provided in Table 6.

**Table 5:** Overall fit indices.

Indicator	CMIN	DF	CMIN/DF	RMSEA	GFI	IFI	CFI	PNFI
Optimal value	—	—	<3	<0.05	>0.90	>0.90	>0.90	>0.90
Measurement result	355.982	278	1.281	0.023	0.950	0.988	0.988	0.811
Meets criteria	—	—	Yes	Yes	Yes	Yes	Yes	Yes

**Table 6:** Path analysis results.

Hypotheses	Path	Std	S.E.	C.R	*p*	Support
H1	ER → FE	0.200	0.053	3.390	***	Yes
H2	INT → FE	0.133	0.056	2.544	0.011	Yes
H3	GQ → FE	0.155	0.058	2.647	0.008	Yes
H4	VN → FE	0.204	0.057	3.551	***	Yes
H5	NC → FE	0.198	0.053	3.304	***	Yes
H6	ER → AW	0.231	0.059	2.133	***	Yes
H7	INT → AW	0.125	0.053	2.333	0.033	Yes
H8	GQ → AW	0.120	0.056	2.400	0.020	Yes
H9	VN → AW	0.139	0.057	2.998	0.016	Yes
H10	NC → AW	0.173	0.058	2.670	0.003	Yes
H11	FE → AW	0.156	0.053	3.916	0.009	Yes

Based on the critical values suggested by Fornell and Larcker (1981) (Fornell and Larcker, 1981) (i.e., CFI > 0.90, RMSEA < 0.06, SRMR < 0.08), the structural model demonstrates a good fit (CMIN/DF = 1.281, CFI = 0.988, RMSEA = 0.023, GFI = 0.950, IFI = 0.988). All paths are statistically significant. Interaction (INT), emotional resonance (ER), visual novelty (VN), generation quality (GQ), and narrative coherence (NC) not only positively impact flow experience (FE) but also positively affect AW. FE significantly influences AW and acts as a mediator. Among them, visual novelty (H9: β = 0.173, *p* < 0.001) has the strongest correlation with AW.

### 4.4 Mediation effect test

The mediation effect test employs the Bootstrap resampling method, where the sample of this study is uniformly resampled 5,000 times with a confidence level of 95%. If the obtained results do not include “0” within the confidence interval, it indicates that the mediation effect of that path is significant, and the mediation effect test is correct; otherwise, it is not. As shown in Table 7, the mediation effect values for all paths are 0.046, 0.047, 0.036, and 0.046, respectively, with confidence intervals excluding 0, indicating that the mediation is established.

**Table 7:** Mediation effect test.

Path	Effect type	Estimate	Lower	Upper	*P*
Interaction ≥ flow theory ≥ AW	Direct effect	0.120	0.022	0.224	0.015
	Indirect effect	0.031	0.007	0.066	0.010
	Total effect	0.151	0.048	0.259	0.002
Emotional resonance ≥ flow theory ≥ AW	Direct effect	0.125	0.012	0.253	0.033
	Indirect effect	0.046	0.015	0.098	0.001
	Total effect	0.171	0.055	0.298	0.005
Visual novelty ≥ flow theory ≥ AW	Direct effect	0.173	0.046	0.307	0.008
	Indirect effect	0.047	0.017	0.099	0.002
	Total effect	0.220	0.098	0.350	0.001
Generation quality ≥ flow theory ≥ AW	Direct effect	0.139	0.011	0.266	0.029
	Indirect effect	0.036	0.009	0.082	0.007
	Total effect	0.174	0.048	0.300	0.005
Coherence of narratives ≥ flow theory ≥ AW	Direct effect	0.156	0.029	0.297	0.018
	Indirect effect	0.046	0.017	0.091	0.001
	Total effect	0.202	0.075	0.339	0.003

### 4.5 ANN results

Although SEM effectively explains the multivariate linear relationships among variables, its predictive power may be limited when dealing with nonlinear and non-compensatory relationships. Therefore, despite the SEM model simplifying the decision-making process, limitations may still exist in capturing complex relationships.

To address these limitations, this study employs the feed-forward back-propagation (FFBP) method to construct an ANN model based on the results of SEM hypothesis testing. FFBP is a neural network model designed to handle complex nonlinear relationships. By adopting the ANN model, the study can more accurately capture and predict nonlinear relationships within the data, thereby improving predictive accuracy and providing more robust support for decision-making.

The ANN model in this study features a structure with two hidden layers, as using two hidden layers enables the modeling of discontinuous functions (Liébana-Cabanillas et al., 2018). The sample was allocated at a 90:10 ratio for training and testing. To minimize error, root mean square error (RMSE) was employed with 10-fold cross-validation.

**Figure 3:**
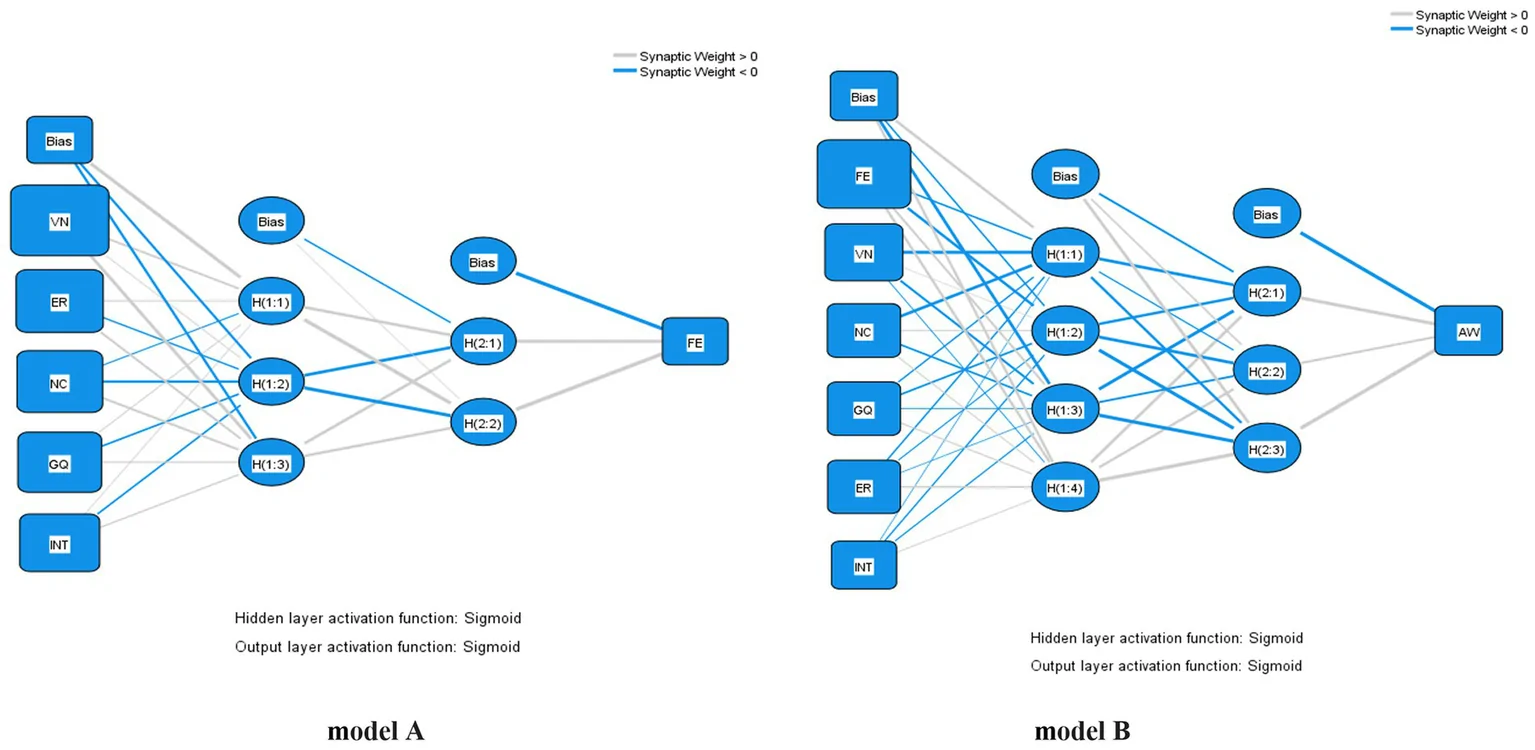
ANN diagram.

In this study, the hidden layer architecture of Model A was automatically generated by SPSS, with three neurons in the first hidden layer and two neurons in the second hidden layer. The hidden layer architecture of Model B was also automatically generated by SPSS, with four neurons in the first hidden layer and three neurons in the second hidden layer. The bias units shown in the network diagram are bias terms and are not counted toward the number of hidden layer neurons. For both models, the sigmoid function was adopted as the activation function in the hidden layers and output layer, in order to capture potential nonlinear relationships among the variables. The sum of squares error function was employed (as shown in Figure 3).

Regarding the stopping rules for model training, explicit stopping criteria were set in the SPSS neural network module. An early stopping strategy implemented in the SPSS neural network module was adopted. Model training was automatically terminated when the training error failed to decrease over one consecutive step or when predefined convergence criteria were met. In addition, predictive performance was evaluated using independent training and testing data to assess model generalizability. Specifically, training was stopped when no decrease in training error was observed over one consecutive step; the maximum training time was set to 15 min; the maximum number of training epochs was automatically computed by SPSS; the minimum relative change in training error was set to 0.0001; and the minimum relative change in the training error ratio was set to 0.001. When the improvement in error during training fell below the above thresholds, the system automatically terminated training. This configuration ensures that training halts once stable convergence is reached, thereby reducing ineffective iterations and the risk of over fitting.

As shown in Table 8, in the testing phase, the mean RMSE of Model A was 0.105, with a standard deviation (SD) of 0.002. The testing error was slightly higher than the training error, but both remained at a low level. This indicates that Model A exhibits good generalization performance with no apparent overfitting, demonstrating favorable predictive performance and stability. For Model B, the mean RMSE in the testing phase was 0.101, with a standard deviation of 0.003, and the testing error was slightly lower than the training error. The consistently low prediction error across both training and testing datasets indicates that overfitting was effectively controlled. Overall, the RMSE values of Model 2 on both the training and testing sets remained at a low level with small fluctuations, indicating that the model exhibited good predictive performance and stability.

**Table 8:** RMSE validation of ANN models.

Neural network	Model A						Model B					
	Inputs: VN, ER, NC, GQ, INT						Inputs: VN, ER, NC, GQ, INT, FE;					
	Output: FE						Output: AW					
	Training			Testing			Training			Testing		
	Samples	SSE	RMSE	Samples	SSE	RMSE	Samples	SSE	RMSE	Samples	SSE	RMSE
ANN1	502	5.368	0.103	44	0.447	0.101	491	4.755	0.098	55	0.572	0.102
ANN2	488	5.387	0.105	58	0.787	0.116	505	5.097	0.100	41	0.397	0.098
ANN3	500	5.628	0.106	46	0.567	0.111	502	4.958	0.099	44	0.361	0.091
ANN4	480	5.147	0.104	66	0.631	0.098	493	5.830	0.109	53	0.432	0.090
ANN5	486	5.181	0.103	60	0.559	0.097	485	4.895	0.100	61	0.553	0.095
ANN6	489	5.679	0.108	57	0.455	0.089	491	4.676	0.098	55	0.623	0.106
ANN7	501	6.084	0.110	45	0.616	0.117	483	4.781	0.099	63	0.625	0.100
ANN8	502	5.465	0.104	44	0.334	0.087	490	4.827	0.099	56	0.557	0.100
ANN9	491	5.254	0.103	55	0.524	0.098	502	5.205	0.102	44	0.296	0.082
ANN10	484	5.344	0.105	62	0.543	0.094	483	4.938	0.101	63	0.450	0.085
MEAN		5.454	0.105		0.546	0.101		4.996	0.101		0.487	0.095
SD		0.281	0.002		0.122	0.011		0.333	0.003		0.115	0.008

As shown in Table 9, the sensitivity analysis reveals the normalized importance ranking of the covariates of the dependent variable in each ANN model. In Model A, the importance ranking of the predictors is as follows: VN (97.24%), ER (90.64%), NC (85.90%), GQ (79.49%), and INT (70.43%). The results indicate that VN is the most important predictor in predicting FE, followed by ER and NC, while the relative contributions of GQ and INT are weaker. In Model B, the importance ranking of the predictors is: FE (96.1%), VN (83.5%), NC (75.6%), GQ (56.4%), ER (50.9%), and INT (50.1%). The results indicate that FE is the most important predictor in predicting AW, followed by VN and NC, while the relative contributions of GQ, ER, and INT are weaker.

**Table 9:** Normalized importance analysis in ANN models.

Neural network	Model A (output: FE)					Model A (output: AW)					
	VN	ER	NC	GQ	INT	FE	VN	NC	GQ	ER	INT
ANN1	0.241	0.201	0.196	0.188	0.174	0.251	0.176	0.154	0.153	0.152	0.115
ANN2	0.252	0.229	0.181	0.216	0.122	0.244	0.246	0.209	0.147	0.081	0.074
ANN3	0.213	0.245	0.189	0.191	0.162	0.256	0.244	0.158	0.123	0.108	0.111
ANN4	0.230	0.216	0.229	0.149	0.176	0.271	0.087	0.189	0.185	0.136	0.133
ANN5	0.207	0.221	0.197	0.190	0.184	0.215	0.192	0.218	0.147	0.102	0.125
ANN6	0.206	0.205	0.200	0.224	0.164	0.247	0.178	0.141	0.145	0.153	0.135
ANN7	0.246	0.200	0.237	0.144	0.173	0.188	0.222	0.185	0.129	0.100	0.177
ANN8	0.256	0.247	0.170	0.176	0.151	0.215	0.178	0.224	0.148	0.123	0.112
ANN9	0.232	0.200	0.233	0.182	0.153	0.227	0.222	0.199	0.081	0.153	0.117
ANN10	0.218	0.181	0.193	0.213	0.195	0.230	0.278	0.138	0.114	0.131	0.108
Average RI	0.230	0.215	0.203	0.187	0.165	0.234	0.202	0.182	0.137	0.124	0.121
Normalized RI (%)	97.24%	90.64%	85.90%	79.49%	70.43%	96.1%	83.5%	75.6%	56.4%	50.9%	50.1%
Ranking	1	2	3	4	5	1	2	3	4	5	6

RI, relative importance.

**Figure 4:**
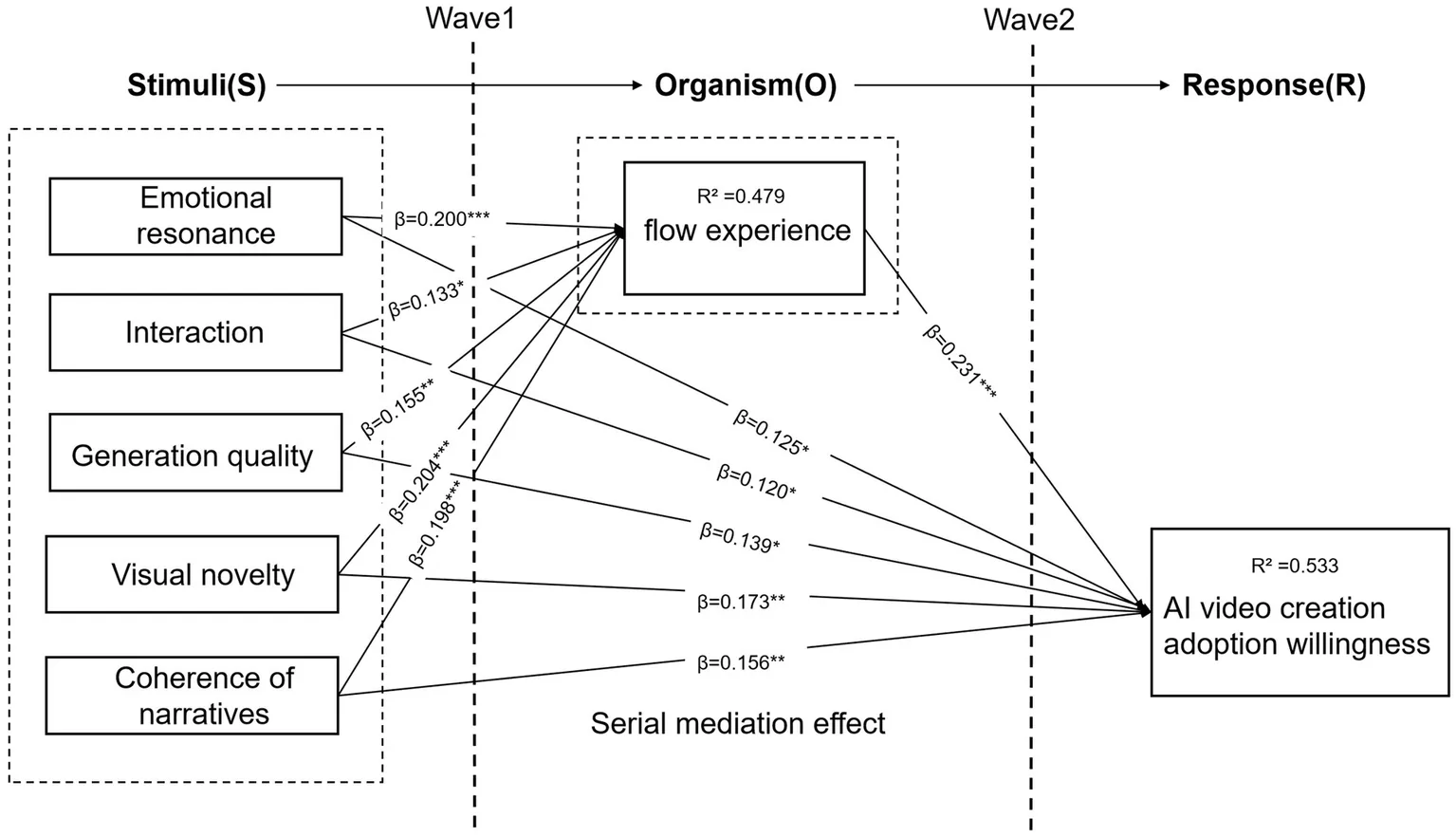
Path coefficient.

The SEM analysis shows (see Figure 4) that all five dimensions of AI video content characteristics significantly and positively influence flow experience and AW, with flow experience (*β* = 0.231, *p* < 0.001) exerting a positive effect on AW. Content characteristics also positively influence AW, with the effect size ranking as follows: visual novelty > narrative coherence > generation quality > emotional resonance > interaction. The mediation test confirms that flow experience plays a significant mediating role between content characteristics and AW. The ANN sensitivity analysis further verifies that visual novelty is the primary predictor of flow experience, while flow experience is the core predictor of AW.

## 5 Discussion

### 5.1 Theoretical contributions

#### 5.1.1 Theoretical contribution 1: extending the application boundary of S-O-R theory into generative AI creation contexts

By conceptualizing AI video creation as a human–AI collaboration process, this study extends the traditional S-O-R theory into the new context of generative media, filling the gap in the application of this theory in the field of AI-generated video content. Rather than replacing the existing framework, this study situates it within the media environment of AI video generation. Unlike prior research primarily grounded in technology adoption or website quality, this study undertakes a contextualized recalibration of the theory in light of the fundamental differences between AI video generation and traditional media consumption across three dimensions: stimulus attributes, organism states, and response characteristics. At the stimulus level, this study decomposes AI video content characteristics into generation quality, emotional resonance, visual novelty, interaction, and narrative coherence, thereby addressing the real-time emergence, dynamic interaction, and unpredictability inherent in AI-generated content. At the organism level, this study defines flow experience as the individual’s internal state, moving beyond the limitation in traditional S-O-R research of reducing the organism variable to a singular affective response. At the response level, this study emphasizes the cumulative and iterative nature of sustained creation willingness. This theoretical extension echoes the research of Pan et al. in the context of digital cultural creative products, similarly validating the effectiveness of the S-O-R framework in explaining users’ behavioral intentions toward digital content (Pan and Zhong, 2026).

Through Bootstrap mediation testing and ANN sensitivity analysis, this study reveals the specific operating mechanism of “stimulus → organism → response”: AI video content characteristics do not directly drive sustained creation willingness; rather, they first induce flow experience, which then, through its “catalytic” effect as an internal state, influences sustained creation willingness. Flow experience plays a significant mediating role between all dimensions of AI video content characteristics and sustained creation willingness. This finding provides cross-domain corroboration with the research conclusions of Hewei (Pathan and Mihir, 2019) and extends them to the AI video generation creation context, which entails greater technical complexity.

#### 5.1.2 Theoretical contribution 2: revealing the differentiated patterns of AI video content characteristics

The SEM path analysis reveals a clear ranking of effect strength: visual novelty > narrative coherence > generation quality > emotional resonance > interaction. This ranking pattern carries 3-fold theoretical significance. First, it challenges the traditional paradigm of technology acceptance. Traditional technology acceptance model (TAM) emphasizes functional value as the primary driver of technology adoption (Davis, 1989), whereas this study demonstrates that, in the context of AI video creation, visual novelty and narrative coherence may surpass functional utility to become the dominant motives. Second, it reveals the “experiential turn” in digital content. The primacy of visual novelty corroborates Coyle’s assertion that “visual vividness enhances user engagement through emotional responses” (Coyle and Thorson, 2001), and this study extends it to the context of AI-generated video content. Third, it highlights the unique role of narrative coherence. As the second most important predictor among content characteristics, narrative coherence underscores the critical role of storytelling in driving users’ AW—AI video platforms are not merely tools, but also infrastructures for story generation and sharing. This further corroborates the findings of Li et al (Pan et al., 2022).

#### 5.1.3 Theoretical contribution 3: identification of nonlinear mechanisms from the dual perspective of SEM–ANN

Through the SEM–ANN hybrid analysis, this study finds that the two methods yield highly consistent rankings of the importance of AI video content characteristics, yet the logic of effect quantification differs fundamentally. SEM path coefficients reflect net linear effects after controlling for other variables, whereas ANN sensitivity indices reveal the compound effects of nonlinear interactions and higher-order thresholds.

Specifically, visual novelty ranks first in both methods, but its ANN importance is significantly higher than its SEM path coefficient, suggesting that when visual stimuli surpass the optimal arousal level, flow experience surges nonlinearly. Emotional resonance and narrative coherence are closely ranked, yet the ANN gap between them is larger, revealing that strong narrative structures can partially compensate for insufficient emotional density, but not vice versa. Interaction ranks last in both methods, but its relative weight in ANN is lower than its SEM standardized coefficient, implying that while moderate interaction promotes immersion, excessive interaction may trigger cognitive overload.

#### 5.1.4 Boundary conditions of the extended S-O-R model in generative AI contexts

Despite the interactive and generative nature of AI video creation, user behavior remains fundamentally driven by the S-O-R mechanism: external system features undergo cognitive and affective processing before generating behavioral intentions. Thus, under specified boundary conditions, the S-O-R framework remains theoretically applicable.

The application of the S-O-R theory in this study is subject to clear boundary conditions. First, this study focuses on active users with over 1 month of experience using AI video platforms; the stimulus–response pathways within the S-O-R framework are therefore predicated on users having already acquired basic technological literacy and do not extend to first-time users of AI video generation. Second, this study examines the flow state that users enter during hands-on AI video creation, rather than the immersive experience of merely viewing AI-generated video content. Accordingly, passive immersion in a purely viewing context falls outside the scope of this study. Third, the five-dimensional content characteristics framework is applicable to AI video platforms centered on prompt-based interaction as the core mechanism; for platforms primarily oriented toward template-based or parameter-driven generation, the relative importance ranking of interaction and narrative coherence may differ. Furthermore, flow experience in AI video generation is conceptualized as a co-creative immersion state arising from real-time interaction with the generative system, rather than passive media consumption.

Taken together, these boundary conditions delineate the scope within which the extended S-O-R model can effectively explain users’ sustained creation willingness in AI video generation environments.

### 5.2 Practical contributions

#### 5.2.1 Differentiated resource allocation pathways for AI video platforms

The variable importance ranking revealed by the ANN sensitivity analysis provides precise guidance for platform resource allocation: the high importance of visual novelty and flow experience indicates that platforms should prioritize investment in visual rendering engine optimization and immersive interface design. By contrast, the relatively lower weight of interaction does not imply that it is unimportant; rather, it suggests that platforms should avoid over-designing complex interactive functions and instead ensure basic interaction fluency and immediate feedback (Cheng and Lu, 2025).

#### 5.2.2 Identifying the nonlinear effect of visual novelty and the optimal arousal mechanism

Although visual novelty is confirmed as the primary predictor of flow experience, its effect may exhibit an “optimal boundary.” The gap between the SEM path coefficient and the ANN sensitivity index suggests that excessive visual stimuli may weaken flow experience. This principle of moderate arousal challenges the linear assumption in traditional technology acceptance research that stronger functions yield better outcomes, thus providing theoretical guidance for the visual design of AI video platforms. AI video platforms should strike a balance between visual appeal and cognitive load, avoiding user attention distraction and immersion disruption caused by excessive rendering.

#### 5.2.3 Implications for AI content governance, Creator education, and ethical standards

Beyond platform-level optimization, the rapid proliferation of AI-generated video content also poses significant challenges for content governance and regulation. Issues such as copyright attribution, synthetic media authenticity, and algorithmic accountability are becoming increasingly prominent. Prior research has emphasized that generative AI systems may amplify risks including misinformation and content manipulation, thus necessitating more robust governance frameworks that extend beyond platform self-regulation to involve multi-stakeholder coordination among policymakers, technology providers, and media platforms (Gao et al., 2026).

These findings also carry important implications for the training and education of content creators in the generative AI era. As AI video generation tools become increasingly prevalent, the role of creators is shifting from manual production to prompt design, content curation, and ethical decision-making. This shift underscores the need to strengthen users’ AI literacy and media ethics education. Educators should therefore focus not only on technical proficiency with generative tools but also on critical evaluation of AI-generated outputs, awareness of potential biases, and responsible content dissemination practices.

Furthermore, establishing industry-wide standards is critical to ensuring the sustainable growth of the generative AI video ecosystem. Standardization efforts may include implementing content labeling mechanisms, synthetic media watermarking, and explicit disclosure requirements for AI-generated content. Such measures can help mitigate ethical risks, enhance user trust, and promote transparency in human–AI media interaction. This study indirectly contributes to this discourse by highlighting the importance of user perceptions and behavioral responses, factors that should be taken into account in the design of future regulatory and industry frameworks.

## 6 Conclusion

This study, based on the S-O-R theoretical framework, examined the influence mechanism of AI video content characteristics on university students’ AW and the mediating role of flow experience. Five content characteristic dimensions—generation quality, emotional resonance, visual novelty, interaction, and narrative coherence—were introduced. The study employed a SEM–ANN hybrid analysis approach for hypothesis testing and predictive analysis, comprehensively evaluating the effects of the five content characteristic dimensions on flow experience and AW. These findings provide practical guidance for optimizing user experience on AI video platforms and enhancing users’ AW.

## 7 Limitations and future work

This study has the following limitations. First, the sample consists of Chinese university students, which may limit the generalizability of the findings. Compared with other demographic groups, university students tend to possess higher levels of digital literacy, greater familiarity with emerging technologies, and more frequent exposure to AI-generated tools—factors that may systematically shape their perceptions, behavioral intentions, and interaction patterns. Caution is therefore warranted when extrapolating the findings to broader populations. Second, the study focuses exclusively on users of a single AI video generation platform, which may introduce platform-specific biases. Different generative AI platforms vary in interface design, algorithmic recommendation mechanisms, and content creation constraints, all of which may influence user behavior and perceptions. Future research should incorporate multiple platforms to enhance cross-platform validity and reduce potential sampling bias. Future studies should also broaden sample diversity by including participants from different age groups, occupational backgrounds, and cultural contexts. In addition, multi-platform comparative studies are recommended to assess whether the observed behavioral patterns hold consistently across different generative AI ecosystems.

Although narrative coherence and visual novelty were adapted from established media research, their manifestation in AI video generation contexts may differ from that in traditional media environments. AI-generated video involves dynamic interaction between user prompts and algorithmic outputs, potentially introducing new dimensions of coherence and novelty that existing measures do not fully capture. Future research should develop context-specific scales to better reflect the distinctive characteristics of AI video content creation.

## Data Availability

The original contributions presented in the study are included in the article/supplementary material, further inquiries can be directed to the corresponding author/s.
